# Insight into Buffalo (*Bubalus bubalis*) RIG1 and MDA5 Receptors: A Comparative Study on dsRNA Recognition and *In-Vitro* Antiviral Response

**DOI:** 10.1371/journal.pone.0089788

**Published:** 2014-02-26

**Authors:** Manvender Singh, Biswajit Brahma, Jitendra Maharana, Mahesh Chandra Patra, Sushil Kumar, Purusottam Mishra, Megha Saini, Bidhan Chandra De, Sourav Mahanty, Tirtha Kumar Datta, Sachinandan De

**Affiliations:** Animal Genomics Lab, Animal Biotechnology Center, National Dairy Research Institute, Karnal, Haryana, India; Faculty of Animal Sciences and Food Engineering, University of São Paulo, Brazil

## Abstract

RIG1 and MDA5 have emerged as important intracellular innate pattern recognition receptors that recognize viral RNA and mediate cellular signals controlling Type I interferon (IFN-I) response. Buffalo RIG1 and MDA5 genes were investigated to understand the mechanism of receptor induced antiviral response. Sequence analysis revealed that RIG1 and MDA5 maintain a domain arrangement that is common in mammals. Critical binding site residues of the receptors are evolutionary conserved among mammals. Molecular dynamics simulations suggested that RIG1 and MDA5 follow a similar, if not identical, dsRNA binding pattern that has been previously reported in human. Moreover, binding free energy calculation revealed that MDA5 had a greater affinity towards dsRNA compared to RIG1. Constitutive expressions of RLR genes were ubiquitous in different tissues without being specific to immune organs. Poly I:C stimulation induced elevated expressions of IFN-β and IFN-stimulated genes (ISGs) through interferon regulatory factors (IRFs) mediated pathway in buffalo foetal fibroblast cells. The present study provides crucial insights into the structure and function of RIG1 and MDA5 receptors in buffalo.

## Introduction

Establishment of an immune response begins with the discrimination between “self” and “non self” components within an organism. To recognize “non self” pathogen-associated molecular patterns (PAMPs), vertebrates have evolved a number of germ-line-encoded receptors called pattern recognition receptors (PRRs). Over the past decades, several types of PRRs have been identified, the recent being cytosolic retinoic acid inducible gene 1 (RIG1)-like helicases (RLHs) including *RIG1* and melanoma differentiation-associated gene 5 (*MDA5*) [1]. *RIG1* and *MDA5* along with another gene *LGP2* (Laboratory of Genetics and Physiology 2) constitutes cytosolic RIG1-like receptors (RLR) family, capable of sensing double stranded RNA (dsRNA) signatures of viruses. RIG1 and MDA5 share sequence homology and transduce downstream signal through a common adaptor called mitochondrial antiviral signaling protein (MAVS) that activates interferon (INF) signaling pathways [2], [3], [4]. RLRs differ from other classes of PRRs *viz.* Toll-like receptors (TLRs) and NOD-like receptors (NLRs), in that these receptors lack repetitive receptor elements such as leucine rich repeats (LRRs) as recognition platform of PAMPs. Instead, they recognize viral dsRNA with a central DExD/H box helicase domain which is characterized by seven conserved motifs (I/Walker A, Ia, II/Walker B, III IV, V and VI) [5], [6]. This conserved “helicase” core of RIG1 and MDA5 is flanked by two tandemly arranged N-terminal caspase activation and recruitment domains (CARDs), and a cysteine-rich C-terminal regulatory domain (CTD) [7]. LGP2 has similar domain architecture as RIG1 and MDA5, but it lacks N-terminal CARDs [8]. In normal cell, CTD plays a role in autoinhibition of RIG1 and MDA5 through intramolecular interactions that mask the CARD signaling [9], [10]. Study on crystal structures of CTD suggests that it plays a prominent role in high affinity binding and selectivity for dsRNA [8], [11]. However, MDA5’s CTD displays lower magnitude of affinity towards dsRNA than that of full-length MDA5 [12]. Thus, despite homology, they differ in molecular mechanisms for dsRNA recognition and play non-redundant function in antiviral immunity [13], [14]. MDA5 detects long duplex RNAs in the genome of double-stranded RNA (dsRNA) viruses or dsRNA replication intermediates of positive-strand viruses, *e.g.* Encephalomyocarditis and vesicular stomatitis viruses [15], [16]. In contrast, RIG1 detects the 5′ triphosphate group (5′ppp) and blunt end of short dsRNA or single strand RNA (ssRNA) hairpins, which are often present in a wide range of negative strand viruses, (*e.g.* influenza A virus) and in some positive and double strand RNA viruses [17], [18]. The role of LGP2 in antiviral response is less clear as compared to RIG1 and MDA5. Since LGP2 lacks N-terminal CARDs, it cannot mediate MAVS based signal transduction. Nonetheless, it is considered to act as a suppressor of RIG1 and MDA5 elicited signaling [19], [20]. Other studies, however, have shown LGP2 as a positive inducer of RIG1 and MDA5 amplifying their responses via a positive feedback mechanism [21], [22].

Buffalo (*Bubalus bubalis*) is an important milk and meat producing animal in many Mediterranean and East Asian countries including India. Besides, Indian buffaloes are well-known for their exceptional ability of disease resistance [23], [24]. However, the genetic basis of disease resistance is still elusive due to paucity of information on molecular mechanism(s) of host-pathogen interaction. Here, we studied the mechanism of viral RNA recognition by buffalo RIG1 and MDA5 receptors through molecular dynamics (MD) simulations. We investigated the expression profile of corresponding genes constitutively, and in response to a stimulus in order to understand the mechanism of RLR mediated antiviral response in buffalo.

## Materials and Methods

### Materials

Taq DNA polymerase, 10X buffer, dNTP were purchased from New England Biolab (MA, USA) and Fermentas Maxima SYBR Green qPCR Master Mix (2X) was obtained from Thermo Fisher Scientific Inc. (PA, USA). Unlabelled goat anti-bovine polyclonal antibodies against RIG1 and MDA5 were obtained from Santa Cruz Biotechnology Inc, (CA, USA) and horseradish peroxidase-labeled rabbit anti-goat antibody was obtained from Sigma-Aldrich (St. Louis, MO, USA). Poly (I:C)/LyoVec HMW (endotoxin level<0.001 EU/µg) was obtained from Invivogen (San Diego, CA). Dulbecco Modified Eagle’s medium (DMEM), Trypsin-EDTA, foetal bovine serum (FBS) and Epidermal Growth Factor (EGF) were obtained from Sigma-Aldrich (St. Louis, MO, USA). L-Glutamine (Glutamax 100x) was purchased from Invitrogen corp., (Carlsbad, CA, USA). Penicillin-G and streptomycin were obtained from Amresco (Solon, OH, USA). Filters (Millex GV. 0.22 µm) were purchased from Millipore Pvt. Ltd., (Billerica, MA, USA). All other reagents were of analytical grade.

### PCR Amplification

All PCR amplifications were performed in 25 µl reaction volume. Each reaction contained 2.5 µl 10X buffer, 200 µM of dNTPs, 0.5 µl of each primers (10 pmol), 0.5 units of Taq DNA polymerase and nuclease free water to bring the total volume to 25 µl. Around 100 ng of cDNA was used as template. The details of primers for different fragments of *RIG1* and *MDA5* genes are summarized in Table 1. Thermal cycling was performed on an ABI Veriti PCR machine where a touchdown protocol with annealing 58°C was followed (Table S1 in File S1). The PCR products were resolved on a 1.5% agarose gel (Figure S1 in File S1). The PCR products were cloned on to pTZ57R/T vector, plasmids were screened using PCR, and positive plasmids were custom sequenced.

**Table 1:** Primers used for amplification of buffalo RIG1 and MDA5 genes.

Gene/Fragment	Primer	Sequences (5′–3′)	Length (bp)	T_m_ (°C)
RIG1Fragment1	Forward	CAGGCATGACGGCAGAGCAGCG	22	62
	Reverse	TTGATAATGAGGGCATCATTGTATTTCCGCA	31	59
RIG1Fragment2	Forward	GTGTTTCAGATGCCAGACAAAGAGGAAGA	29	60
	Reverse	TCTTCCTCTGCCTCTGGTCTGGATCAT	27	61
RIG1Fragment3	Forward	GTCGCCGATGAAGGCATTGACATTGC	26	61
	Reverse	CCTGAGCCCAAGGGGACATTTCTGC	25	63
MDA5 Fragment1	Forward	AGATGTCGTCGGACGGGAGTTCAACAGA	28	63
	Reverse	CTGAATCACTTCCCATGGTGCCTGAATC	28	61
MDA5 Fragment2	Forward	GTTTGGCAGAAGGAAGTGTCAGCTGCT	27	61
	Reverse	CCTCATTGTACTTCCTCAGATGTTCTGCAC	30	62
MDA5 Fragment3	Forward	AGCTGCAAAAGAAGGAAATCGCAAAGATCGTG	33	62
	Reverse	GAAATCTTGAATCAAATGTCAGTCCTCATCAC	32	59

### Immunohistochemistry (IHC)

Spleen, tonsil and thyroid samples were collected from Municipal Corporation of Delhi (MCD) Slaughter House, Ghazipur, New Delhi, India with permission for research use. The samples were fixed with 10% neutral-buffered formalin and embedded in paraffin. For antigen retrieval, thin sections of tissues (∼3 µm) were deparaffinized with xylene and rehydrated by washing with ethanol (95%) and MiliQ water. Non-specific sites were blocked by overnight incubation of sections with a blocking reagent (1% BSA, 10% FBS-PBS) at 4°C. Tissue sections were then stained with either anti-RIG1 (1∶100 dilution) or anti-MDA5 (1∶150 dilution) for 1 h at room temperature. Slides were washed three times with Tris-buffered saline (pH 7.6) supplemented with 0.05% Tween-20. Thereafter, the slides were treated with horseradish peroxidase-labeled rabbit anti-goat antibody for 1 h and washed as described above. The slides were then mounted with DAB (3, 3′-diaminobenzidine) substrate chromogen solution for 5 minutes. Counter-staining was performed with Mayer’s haematoxylin. The slides were rinsed with water, dried and visualized.

### Poly I:C Treatment of Foetal Fibroblasts

Gravid uteri (2–3 months of pregnancy) were obtained from abattoir house, New Delhi Municipal Corporation. For poly I:C treatment, buffalo foetal fibroblast cells at about 70% confluence were harvested and seeded at a concentration of 1×10^5^cells/ml into four well culture plates (Text S1 in File S1). The cells were cultured in DMEM (10% FBS) for 24 h at 37°C. After 24 h, serum containing medium was decanted and cells were washed twice with Dulbecco’s phosphate buffer saline (DPBS) solution. Cells were then maintained with serum free DMEM medium at 37°C for 6 h. The medium was subsequently discarded and cells were maintained either with serum free DMEM medium (control group) or serum free DMEM medium containing 2 µg/ml of Poly (I:C)/LyoVec (treatment group). The experiment was performed with four replicates for each treatment or control group.

### Real Time PCR (qRT-PCR)

Real time PCR primers were designed by aligning gene sequences of several mammals including cow, pig, mouse, buffalo, and human (Table 2). Equal amount of RNA (quantified by Qubit fluorometer, Invitrogen), wherever applicable, were used for cDNA preparation (Superscript III cDNA synthesis kit; Invitrogen). All qRT-PCR reactions were conducted on a Light Cycler 480 II Real-Time PCR machine (Roche Diagnostics, USA). Each reaction consisted of 2 µl cDNA template, 5 µl of 2X SYBR Green PCR Master Mix, 0.25 µl each of forward and reverse primers (10 pmol/µl) and nuclease free water for a final volume of 10 µl. Each sample was run in duplicate. Analysis of real-time PCR (qRT-PCR) was performed by delta-delta-Ct (ΔΔCt) method [25].

**Table 2:** Primers used for mRNA quantitation of different genes by Real time PCR (qRT-PCR).

Gene	Primer	Sequence (5′-3′)	T_m_ (°C)	Product Size (bp)
*RIG1*	Forward	CTTGCAAGAGGAATACCACTTAAACCCAGAGAC	63	150
	Reverse	TTCTGCCACGTCCAGTCAATATGCCAGGTTT	63	
*MDA5*	Forward	TCTGCTTATCGCTACCACAGTGGCAGA	61	135
	Reverse	TGCTCTCATCAGCTCTGGCTCGACC	63	
*LGP2*	Forward	CCACCACGTCAATGTGAACCCCAACTTC	63	176
	Reverse	TGAGCACTGGCAGCTTCACTGACTTGTAGAT	63	
*MAVS*	Forward	CATCAGGAGCAAGACACAGAACTGGGCA	63	115
	Reverse	AACGGGCCAAGGGCTGGAAGGAGACAC	66	
*IRF3*	Forward	GATACTGCCCTGGCTGATATCTCAGCTG	63	235
	Reverse	GTTCAGGGCAGACCGGAAATTCCTCTTC	63	
*IRF7*	Forward	GCCTCCTGGAAAACCAACTTCCGCTG	63	340
	Reverse	GCAGATGGTCCTCCAAGCAGCTCTG	63	
*IFNβ*	Forward	CACCACAGCTCTTTCCAGGAGCTACA	61	168
	Reverse	GGAACTGCTGTTCTTGCTTCATCTCCTC	61	
*ISG15*	Forward	AGATCAATGTGCCTGCTTTCCAGCAGCG	63	162
	Reverse	GACCCTTGTCGTTCCTCACCAGGATG	61	
*ISG54*	Forward	AAGTGCACGGCAATCATGAGTGAGACCA	61	94
	Reverse	CCTCTACCAAGTTCCAGGTGAAATGGC	61	
*ISG56*	Forward	CTATGTGAAACACCTGAGAGGCCAGAATGA	62	132
	Reverse	CAGGCATAGTTGCCCCAGGTAACCAG	63	

### Molecular Dynamics Simulations

The initial structures of RIG1 and MDA5 were obtained using homology modeling (Text S2 in File S1). Molecular dynamics (MD) simulations of modeled RIG1 and MDA5 were carried out in GROMACS 4.5.5 [26] using Charmm27 all-atom force field. All the structures were hydrated using SPC216 water molecules in two separate cubic boxes with a distance of 10 Å between the proteins and box edges. Physiological ionic strengths (0.15 M) of counter ions were added to neutralize total charge of the simulation systems. The electroneutralized systems were subjected to steepest descent energy minimization to remove steric conflicts between atoms. The minimized models were then subjected to MD simulations using NPT ensemble with constraints on the backbone heavy atoms for 100 ps, keeping the number of particles (N), the system pressure (P), and the temperature (T) constant. The temperature of the systems was coupled to 300 K via Berendsen temperature coupling scheme, and the pressure of the systems were maintained at 1 bar using Parrinello–Rahman algorithm. Finally, the models of RIG1, and MDA5 were subjected to 2 ns MD simulations without any constraints on the backbone atoms. Trajectory and subsequent data analyses were performed using VMD 1.9.1 [27], Grace 5.1.23 (http://plasma-gate.weizmann.ac.il/Grace/), and Pymol1.3rc2 (The PyMOL Molecular Graphics System, Version 1.3 Schrödinger, LLC) software. Principal component analysis of MD trajectories was performed using ‘g_covar’ and ‘g_anaeig’ programs of GROMACS (Text S2 in File S1).

### Binding Free Energy Calculation

The binding free energies (ΔG_bind_) of RIG1-dsRNA and MDA5-dsRNA were calculated with GMXAPBS tool [28]. The detailed procedure is outlined in Text S2 in File S1.

### Statistical Analysis

All statistical analyses were carried out using SYSTAT 13.1 software (SYSTAT Software Inc.) Analysis of variance (ANOVA) was used to test between groups and hour intervals. Fischer’s restricted least significant differences criterion was used to maintain the *a priori* type I error rate of 0.05.

## Results

### Sequence Analysis of Buffalo RIG1 and MDA5 Genes

**Figure 1:**
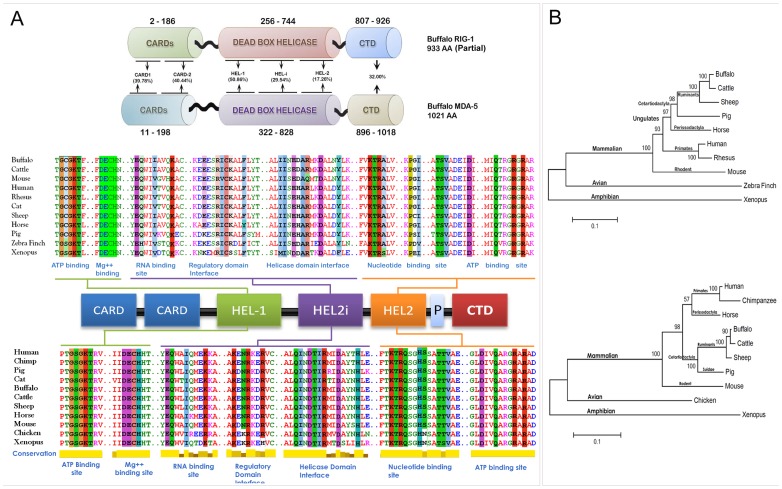
Amino acid sequence analysis of RIG1 and MDA5 receptors. (A) Sequence comparison of functional domains and binding residues of RIG1 and MDA5. Multiple sequence alignment was performed by MAFFT web server and binding site residues (highlighted) were identified by DELTA-BLAST and CDD database of NCBI. (B) The evolutionary history was inferred by using the Maximum Likelihood method of MEGA5 based on the JTT matrix-based model. The trees with the highest log likelihood are shown. Initial tree(s) for the heuristic search were obtained automatically by applying Neighbor-Join and BioNJ algorithms to a matrix of pairwise distances estimated using a JTT model, and then selecting the topology with superior log likelihood value. Sequences used for analysis are provided in Table S2 in File S1.

The amino acid sequences of buffalo RIG1 and MDA5 were translated from respective nucleotide sequences identified in this study (GenBank Accession ID: KF517376 and KF517377; Text S3 in File S1). The putative conserved domains and critical binding site residues within RIG1 and MDA5 were identified using DELTA BLAST and Conserved Domain Database (CDD) of NCBI. Comparison of amino acid conservation among different domains of buffalo RIG1 and MDA5 sequences revealed that CARD, helicase, and CTDs of these genes share 31.1, 34.9 and 21.5% homology, respectively (Figure 1). Putative conserved domains along with conservation of critical binding site residues among buffalo RIG1, MDA5 and different model organism has been shown in Figure 1A. From Figure 1A it is clear that critical binding site residues of both RIG1 and MDA5 are conserved across mammals. Moreover, the residues responsible for ATP, Mg^++^, and RNA binding are almost identical. Phylogenetic trees constructed by maximum likelihood method (MEGA5.2 software) [29] revealed a similar pattern of evolution for RIG1 and MDA5 in different model organisms except perrisodactyla (horse), where, RIG1 was clustered with cetartiodactyla and MDA5 with primates (Figure 1B).

### Structural Analysis of RIG1 and MDA5 Proteins

The modeled buffalo RIG1 and MDA5 exhibits characteristic tertiary folds containing HEL-1, HEL-2, HEL-2i, and the CTD (Figure S2 in File S1). The stereochemical qualities of the modeled proteins were satisfactory, indicating that the models could be used in simulation studies (Figure S3 in File S1; Table S3 in File S1).

**Figure 2:**
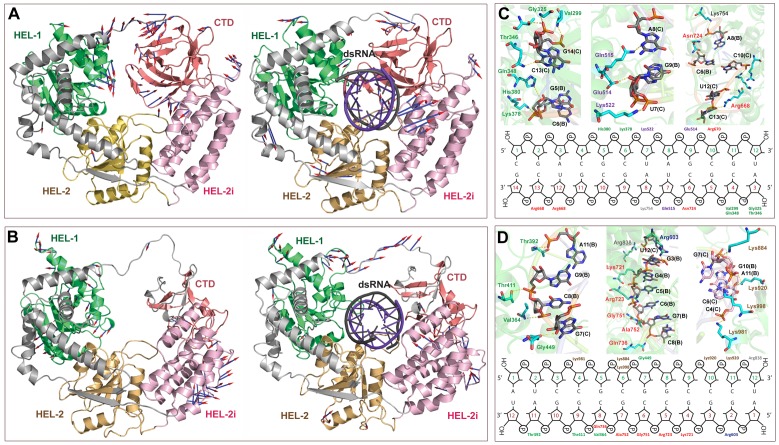
Structural movements of RIG1 and MDA5 receptors during MD simulation. Porcupine plots showing structural movements of (A) RIG1 and (B) MDA5 receptors during MD simulations. The structural movements are shown for both RNA-bound and free proteins. The docked dsRNA molecule has been colored in violet color. Arrow heads indicate direction of motion and length of the arrows specifies the extent of displacement. The figure also shows the schematic representation of intermolecular interactions (C) RIG1 and (D) MDA5 with dsRNA after MD simulations.

**Figure 3:**
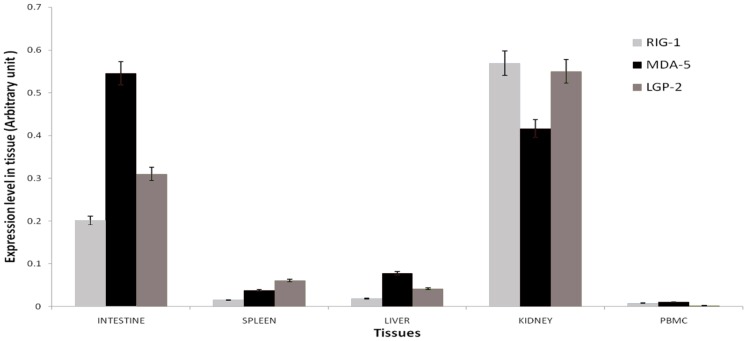
Constitutive mRNA expression levels of buffalo RLR (*RIG1, MDA5* and *LGP2*) genes in different tissues. Relative abundance of mRNA was measured by qRT-PCR using *RPS-18* as housekeeping control.

MD simulations were performed on free and dsRNA bound RIG1 and MDA5 to analyze their structural organization and intermolecular interactions. Analysis of MD trajectories revealed that the backbone as well as the individual residues of RNA bound proteins fluctuate less compared to free proteins (Figure S4 in File S1). This observation prompted us to study the global motions of individual domains during MD simulation. Principal component analysis (PCA) revealed that CTD of free RIG1 shows maximum displacements (Figure 2A). However, CTD of RNA bound RIG1 was comparatively rigid. Further, it was found that HEL-2i domain of both free and RNA bound proteins display movements (Figure 2A). As can be seen from Figure 3B, all four domains of RIG1, *i.e.* HEL-1, HEL-2, HEL-2i, and CTD showed rotations and translations upon RNA binding. Similarly, free as well as RNA bound MDA5 showed noticeable motions of individual domain (Figure 2B). In addition, the V shaped linker of RNA bound proteins showed major displacements as compared to unbound proteins. Collectively, all four domains were found to be involved in dsRNA recognition.

The intermolecular contacts of buffalo RIG1 and MDA5 with dsRNA was identified and compared with those of human (Figure 2C–D; Table 3). We found that buffalo and human share a similar pattern of dsRNA recognition mechanism. In buffalo and human RIG1, positively charged residues were primarily involved in dsRNA recognition. Additionally, hydrophobic and polar residues were found to interact with dsRNA. In contrast, polar and hydrophobic residues of MDA5 were mostly involved in dsRNA binding before MD simulation. However, the number of Arginine around dsRNA increased considerably after MD simulation (Table 3). On the contrary, MDA5 displayed greater number of Lysine around dsRNA, suggesting positively charged residues are indispensible for viral RNA recognition by buffalo RIG1 and MDA5.

**Table 3:** Comparative intermolecular interaction of RIG1 and MDA5 (human and buffalo) with dsRNA.

RIG1						MDA5					
Human		Buffalo				Human		Buffalo			
RIG1	dsRNA	Before simulation		After simulation		MDA5	dsRNA	Before simulation		After simulation	
		RIG1	dsRNA	RIG1	dsRNA			MDA5	dsRNA	MDA5	dsRNA
Ile300	C13	Val299	C13	Val299	C13	Lys365	C8	Val364	C8	Val364	C8
Gly326	G14	Gly325	G14	Gly325	G14	Val366	C8	Gly390	G9	Thr392	A11
Thr347	C13	Gln511	A8	Thr346	G14	Asn419	G9	Thr411	G9	Thr411	G9
Lys379	C6	Arg641	C10	Gln348	C13	Lys450	G8	Arg603	G3	Arg603	G3
Arg637	C10	Arg668	U12	Lys378	C6	Gln584	G2	Thr722	C5	Cys721	G4
Arg637	G11	Arg670	C10	His380	G5	Lys587	G2	Arg723	C5	Arg723	C5 C6
Gly663	G11	His834	C1	Glu514	A8	Arg605	G3	Arg723	C6	Arg723	C5 C6
Arg664	U12	Trp912	G2	Gln515	G9	Arg728	C5	Gly751	C6	Gly751	C6
His830	C1			Lys522	U7	Ala757	G7	Ala752	G7	Ala752	G7
Glu510	A8			Arg641	C10	Gln771	G7	Lys998	C6	Gln763	C8
				Arg668	U12	Asn812	C9	Val1000	G5	Arg838	U12
				Arg668	C13	His927	C3	His922	C3	Lys884	G7
				Arg670	C10	Lys1002	C6	Met921	C4	Lys920	G10
				Asn724	C6	Val1004	G5	Asn578	U12	Lys920	A11
				Cys754	A8			Gln913	C8	Lys981	C4
								Glu919	A11	Lys998	C6
										Gly449	G7

The overall binding free energy (ΔG_bind_) of MDA5−dsRNA complex was found to be greater than that of the RIG1−dsRNA complex (Table 4). The affinity of dsRNA towards the receptors differs only at the electrostatic (ΔG_coul_) and polar contributions (ΔG_polar_). The ΔG_coul_ and ΔG_polar_ of MDA5−dsRNA complex were calculated to be greater than those of RIG1−dsRNA complex. Hence, it appears that polar and electrostatic interactions play dominant role for dsRNA recognition by RIG1 and MDA5.

**Table 4:** MM/PBSA binding free energies (kJ/mol) of RIG1/dsRNA and MDA5/dsRNA complexes.

Energy component	RIG1/dsRNA (kJ/mol)	MDA5/dsRNA (kJ/mol)
ΔG_coul_	−23858.9 (6.0)*	−26288.4 (5.1)
ΔG_ps_	28789.7 (6.2)	32055.2 (5.6)
ΔG_polar_	4930.8	5766.8
ΔG_vdW_	−3431.18 (1.2)	−3487.69 (1.8)
ΔG_nps_	−96.0808 (0.3)	−93.3481 (0.5)
ΔG_nonpolar_	−3527.26	−3581.04
ΔG_bind_	−1403.5 (3.7)	−2185.78 (3.5)

*Standard errors are indicated in parenthesis.

ΔG_coul_ = Electrostatic energy.

ΔG_ps_ = Polar solvation energy.

ΔG_polar_ = Polar contribution (ΔG_coul_+ΔG_ps_).

ΔG_vdW_ = van der Waals energy.

ΔG_nps_ = Nonpolar solvation energy.

ΔG_nonpolar_ = Nonpolar contribution (ΔG_nps_+ΔG_vdW_).

ΔG_bind_ = Overall binding energy.

### Constitutive Expression of RLR Genes in Different Tissues

**Figure 4:**
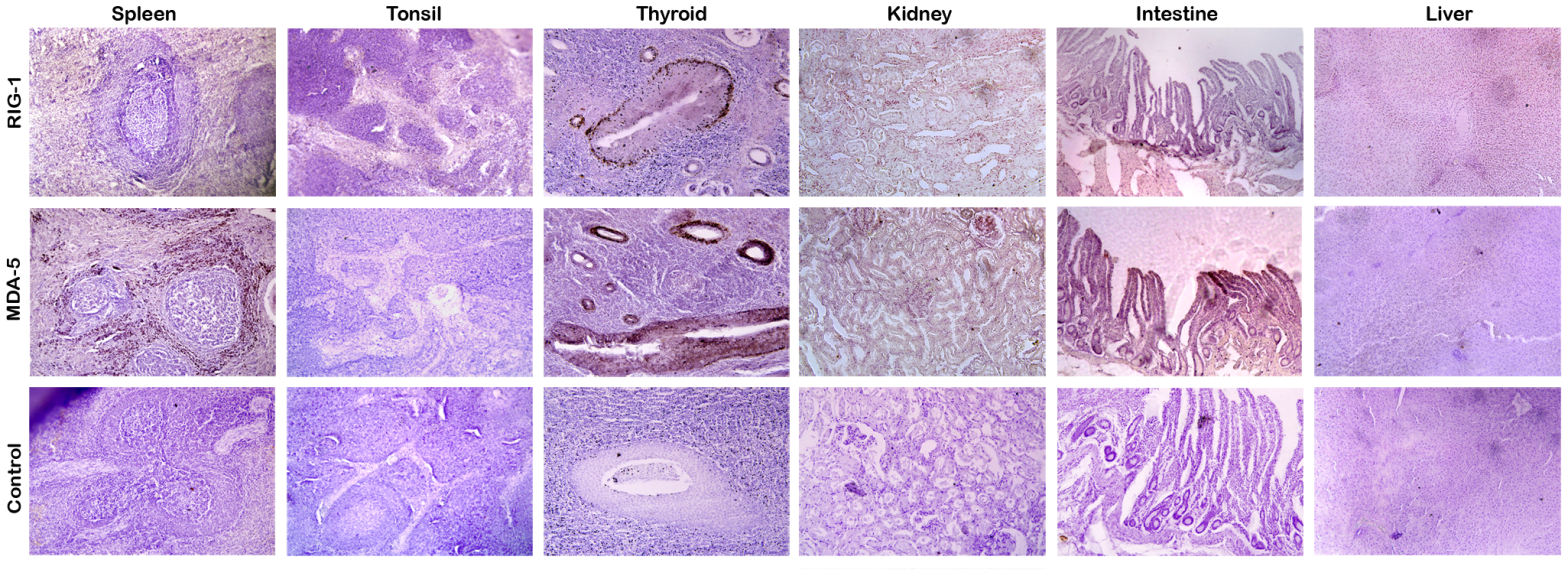
Constitutive protein expression levels of buffalo RIG1 and MDA5 in different tissues. Imuunohistochemical localization of RIG1 and MDA5 were detected in thin tissue sections using specific antibodies with DAB (3,3′-Diaminobenzidine) as substrate. Counterstaining was performed with Mayer’s haematoxylin.

Basal expression of the three buffalo RLR genes (RIG1, MDA5, and LGP-2) was studied in liver, spleen, intestine, kidney, and PBMC using qRT-PCR with RPS-18 (40S ribosomal protein S18) as the internal reference gene. It was found that the RLR genes were ubiquitously expressed in every tissue tested (Figure 3). The expression levels of RLR genes were comparatively higher in kidney and intestine than in spleen and PBMC. The ubiquitous nature of RLR expression of was also reflected at protein level. Immunohistochemical localization of RLR proteins were detected in primary immune organs (spleen and tonsil) as well as in thyroid, kidney, which are not directly involved in primary immune response (Figure 4). Marked staining can be seen for RIG1 and MDA5 across the red pulp area of spleen, follicular epithelial cells and parafollicular cells of thyroid tissue, renal tubular epithelial cells, mucosal and sub-mucosal layers of intestine. A lower expression of RIG1 and MDA5 was observed in crypts of tonsil and in hepatocytes. Negative control sections with an isotypic IgG antibody were run in parallel; no staining was observed in any of these control sections. The primary antibodies used for RIG1 and MDA5 were specific, as revealed from western blot analysis (Figure S5 in File S1).

### Responses of RLR and Associated Genes to poly I:C

Following poly I:C exposure, mRNA expression pattern of RLR genes, downstream adapter genes (MAVS, IRF-3 and IRF-7) and the effector genes (IFN-B and ISGs) were analyzed in foetal fibroblast cells. Fibroblast cells provide an obvious advantage for studying RLR mediated antiviral response, because TLR system for viral recognition is dispensable in this cell type [30], [31] Primary culture was chosen over established immortalized cell lines because the later may have multiple mutations that can affect the permissiveness and responsiveness to viral infections [32]. All experiments were carried out on fibroblasts with less than 10 passages.

### Expression Patterns of RLR Genes

**Figure 5:**
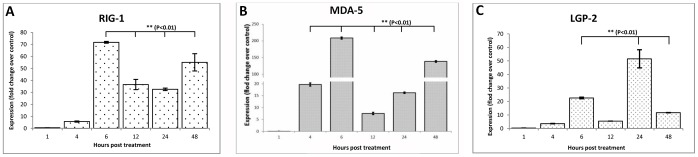
Poly I:C induced expression levels of RLR genes in buffalo foetal fibroblast cells over different time intervals. Relative fold change in mRNA of (A) RIG1 (B) MDA5 and (C) LGP2 over control has been shown.

As shown in Figure 5, RLR genes showed no change in expression after 1 h post treatment, but *RIG1* and *MDA5* genes were up-regulated (p<0.01) after 4 h post treatment. Maximum expression of these genes was seen during 6 h post treatment. Afterwards, the expression of *RIG1* was sustained at a higher level until 48 h. The level of *MDA5* dropped transiently during 12 h, but increased again during subsequent intervals of experiment. The expression pattern of *LGP2* followed a slightly different pattern (Figure 5C). The mRNA expression level of *LGP2* was significantly higher (p<0.01) during 6 h and was maximum at 24 h after poly I:C treatment. It appeared that the amplitude of response was higher for *MDA5*, while *RIG1* maintained a sustained response following poly I:C treatment.

### Expression Patterns of MAVS, IRF-3, and IRF-7

**Figure 6:**
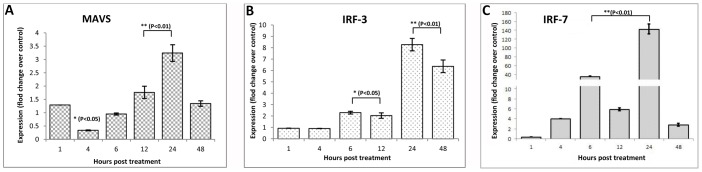
Poly I:C induced expression levels of RLR adapter genes in buffalo foetal fibroblast cells over different time intervals. Relative fold change in mRNA of (A) MAVS (B) IRF-3 and (C) IRF-7 over control has been shown.

The expression pattern of MAVS showed an unusual trend following poly I:C treatment (Figure 6A). The mRNA expression level was significantly lower (p<0.05) during 4 h post-treatment. The expression level increased during subsequent hours and was significantly higher (p<0.01) during 12–24 h post-treatment. Again during 48 h the expression dropped to basal level. The induction of both IRF-3 and IRF-7 following poly I:C treatment was evident in the present experiment (Figure 6B–C). The mRNA levels of both IRF-3 and IRF-7 were significantly higher during 6 h post treatment, though amplitude of response was much higher for IRF-7. The expression level showed a marked increase (p<0.01) during 24 h for both IRF-3 and IRF-7. In general, the expression pattern of IRF-3 and IRF-7 followed a similar trend until 24 h, after which the level of IRF-3 was constant until 48 h, but the level of IRF-7 dropped close to basal level.

### Expression Patterns of IFN-β and ISGs

**Figure 7:**
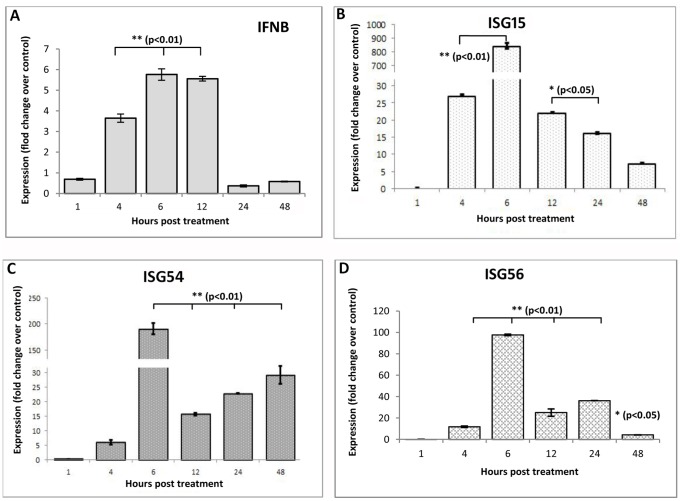
Poly I:C induced expression levels of IFN-β and ISGs in buffalo foetal fibroblast cells over different time intervals. Relative fold change in mRNA of (A) IFN-β (B) ISG15 (C) ISG54 and (D) ISG56 over control has been shown.

Poly I:C was found to induce IFN-β response in bovine foetal fibroblast cells. As shown in Figure 7A, IFN-β expression started increasing after 4 h post-treatment. Expression of IFN-β mRNA was highest during 6 h, higher level was maintained up to 12 h, and finally returned to basal level at 24 h post treatment. Induction of Type-I IFN response leads to transcriptional induction of >300 IFN-stimulated genes (ISGs) that have been shown to function as antiviral effectors. This was evident in the present experiment as well. As early as 4 h post-treatment, the transcript level of all ISGs (ISG-15, ISG-54 and ISG-56) increased (Figure 7B–D). At 6 h post treatment, a sharp increase in the expression level of all three ISGs was observed, the level of ISG-15 being the maximum. It was found that during subsequent time period the levels of all ISGs were sustained at higher concentration.

## Discussion

Over the last decade, studies have highlighted the biological role and importance of RLRs in antiviral immunity [7]. However, compared to other mammalian, avian, amphibian, and fish [33]–[35] species, bovine RLRs have been poorly studied. Here, we reported the evidence of functional RIG1 and MDA5 genes in buffalo genome. Domain analysis revealed that buffalo RIG1 and MDA5 receptors have common domain architecture as in other mammals. It is noteworthy that only MDA5, but no RIG1 ortholog, is present in all teleosts**,** the first chordates to be evolved. This indicates that MDA5 might precede RIG1 in the evolutionary history. It has been postulated that the functional domains present in MDA5 and RIG1 have evolved independently, and most probably, not by a simple gene-duplication event [36]. The helicase domains might have been evolved from a eukaryotic ancestor gene, common to DICER and DEAD/DEAH box RNA helicase-I of MDA5 and RIG1, but the emergence of distinct CARD domains might have happened by domain grafting [36], [37]. However, identification of RIG1-like genes in the genomes of sea anemone (*Nematostella vectensis*) and sea urchin [38] suggests that RIG1 might have emerged from invertebrates, but gradually got lost in some fish lineages [37]. While the origin of RLR genes remains inconclusive, our sequence analysis indicated that the DEAD/DEAH box RNA helicase (HEL-1 and HEL-2) of these two genes may share a common ancestor as revealed by the conservation of critical binding site residues *viz.* ATP binding site [G-(C/S)-G-K-T and D-Q-R-R], Mg^++^ binding site [D-E-C-H], and nucleotide binding site [K-T-R-(A/Q)-(P/H)-(I/V/S/N)-T-(S/T)-V]. Despite high sequence identity at RNA binding sites (E-Q/H-I/V-K) of RIG1 and MDA5, substantial differences were observed within HEL-2i of both the proteins. However, the residues of HEL-2i domain were conserved in both RIG1 and MDA5 across other mammalian species considered for this study. Our result suggests that irrespective of their point of phylogenetic diversion, critical binding site residues of RIG1 and MDA5 are extremely conserved. This indicates that RNA binding pattern, a critical step for inducing antiviral innate immune responses, must be similar throughout the mammalian species.

High resolution crystal structures of RIG1 and MDA5 with or without viral RNA have provided a better understanding of RNA-induced receptor activation [5], [10], [39]–[42]. However, these studies were carried out on human, mouse, or duck models, but information on RNA mediated receptor activation in bovine is sparse. Our simulation studies elucidated a network of interactions between dsRNA and four domains of buffalo RIG1 and MDA5. This mode of interaction was consistent with those observed in the crystallographic structures [5]–[6], [39]. In both the receptors, positively charged residues were involved in providing affinity to dsRNA, as observed by the binding free energy calculation. Analysis of binding free energy terms indicated that MDA5 binds dsRNA with a higher affinity than RIG1 does. We found the number of positively charged amino acids that interacted with dsRNA were higher in MDA5 than RIG1. This could be the reason of difference in net binding free energy and affinity of dsRNA-receptor complexes.

The constitutive expression levels of the RLR genes were not restricted to the immune organs. Instead, organs like kidney and intestine showed a higher level of constitutive expression. This ubiquitous expression pattern of RLRs has also been observed in human, mice, and fishes [43], [44]. Higher expression level in intestine is consistent with these receptors’ role in maintaining the intestinal epithelial barrier that prevents entry of many viruses. Taken together, these findings suggest that many cell-types have the potential for RLR mediated response against viral invasion.

The inductive expression patterns of RIG1 and MDA5 in response to poly I:C were different. The induction of MDA5 started early, amplitude of expression was higher, but expression level varied over time. However, RIG1 was induced later, but its expression level was sustained during different hours of experiment. The higher amplitude of MDA5 induction could be attributed to its greater affinity for dsRNA than RIG1, as revealed from our MD simulation studies. It seems that LGP2 negatively regulated both RIG1 and MDA5, as its expression pattern followed a reverse trend compared to RIG1 and MDA5. The role of LGP2 in RLR mediated antiviral responses remains controversial as it has been reported both as a suppressor [19], [20] as well as a positive inducer [21], [22] of RIG1 and MDA5 elicited signaling. LGP2 lacks N-terminal CARD regions, essential for MAVS mediated signaling, and keeps a check on RIG1 and MDA5 mediated overproduction of IFNs [45]. Our results suggested that while RIG1, MDA5, and LGP2 are simultaneously involved in viral RNA recognition and the role of LGP2 might be regulatory in nature.

After sensing dsRNA, RIG1 and MDA5 interact with CARD domain of MAVS, leading to downstream activation of IRF-3, IRF-7 and IFN-β production [2], [46]. However, we found the mRNA expression of MAVS was significantly (p<.05) downregulated during 4 h. RLR activation can induce a selective proteasomal degradation of MAVS, which is essential for downstream signaling [47]. Also, poly I:C can significantly downregulate MAVS expression in early hours of induction by rapidly decaying the MAVS mRNA [48]. Therefore, the initial downregulation of MAVS could be poly I:C mediated degradation of mRNA. Subsequent increase may be a consequence of cells’ demand of MAVS for RLR signaling. MAVS activation causes homo and hetero dimerization of IRF3 and IRF7, which induce type I IFNs. IRF3 is constitutively expressed and involved in immediate antiviral responses, while IRF7 is expressed in a limited fashion and is induced after IFNβ production is initiated [49]. Consistent with these findings, the present study also showed delayed induction of IRFs. Expression of IRF7 was temporal with high amplitude, but IRF-3 consistently showed a higher expression level after induction.

Following poly I:C treatment, buffalo foetal fibroblast cells triggered an elevated IFN-β expression and transcription of IFN-stimulated genes (ISGs). The expression pattern of IFN-β was consistent with previous studies [50]. It is worth noting that despite negligible expression level of IFN-β during 24–48 h, the expression levels of ISG15 and ISG54 continued to be high. This could be explained as activated homo- and heterodimers of IRF3 and IRF7 are able to bind IFN-stimulated response elements (ISRE) at the promoter regions of ISGs and can direct IFN-independent activation of ISGs [51]. Among ISGs, ISG56 can negatively regulate ISG15, ISG56, IRF3 and IFN-β by interacting with the adapter protein MITA to disrupt virus-induced IRF3 activation and cellular antiviral responses [52]. In agreement to this finding, the expression pattern of ISGs in our study suggests that ISG56 could be a negative regulator of ISG15 and ISG54, besides inhibiting the expression of IFN-β.

## Conclusion

In conclusion, functional RIG1 and MDA5 genes of buffalo were found to follow similar pattern of domain organization as in other mammals. MD simulation studies suggested that despite striking homology at binding site residues, RIG1 and MDA5 receptors have different affinity towards dsRNA. Buffalo RLR genes are ubiquitously expressed in different tissues, irrespective of function of the tissue as immune organ. Poly I:C can induce an antiviral response in buffalo foetal fibroblast cells through RLR mediated pathway.

## Supporting Information

File S1 **Supporting Text, Tables, and Figures. Text S1:** Methodology for isolation and culture of buffalo foetal fibroblast cells. **Text S2:** Procedures for model construction and simulation analysis. **Text S3:** Deduced amino acid sequence of buffalo RIG1 and MDA5. **Table S1:** PCR cycling parameters used for the amplification of RLR genes. **Table S2:** Sequences (NCBI Accession numbers) used for phylogenetic analysis. **Table S3:** Comparative analysis of the stereo-chemical parameters of RIG1, MDA5 and their corresponding templates. **Figure S1:** Agarose gel electrophoresis of amplified products of different fragments of buffalo RIG1 and MDA5 genes. **Figure S2:** Overall tertiary structure of helicase and C-terminal domains of (A) RIG1 and (B) MDA5 proteins of buffalo. The helicases and C terminal domains of buffalo RIG1 and MDA5 were modeled based on the X-Ray crystallographic structures of human RIG1 and MDA5 (termed as templates). The pair-wise alignment of buffalo and human sequences showed 82 and 84 percent sequence identities for RIG1 and MDA5, respectively. Due to this striking homology between target and template proteins, the built models retained all the key structural features of the templates that included HEL-1, HEL-2, HEL-2i, and the C-terminal domains. **Figure S3:** Ramachandran plot of buffalo (A) RIG1 and (B) MDA5 receptors. Stereochemical qualities of modeled proteins were found to be highly comparable to those of templates, indicating quality of the models were reasonably good to carry out further studies. **Figure S4:** Stability parameters of RIG1 and MDA5 receptors as a function of simulation time. (A) RMSD of RIG1. (B) RMSD of MDA5. (C) RMSF of RIG1. (D) RMSF of MDA5. In each panel, red color indicates stability parameters of dsRNA bound receptors and black color denotes receptors without dsRNA. **Figure S5:** Western analysis of intestinal tissue to determine specificity and cross reactivity of primary antibodies to RIG1 and MDA5. Dilution of primary antibodies 1∶500; Dilution of secondary anti goat IgG 1∶50000. The chemiluminescence was detected on x-ray films using Immobilon Western Chemiluminescent HRP (Millipore Corporation, MA, USA) as substrate.
(PDF)

## References

[1] Yoneyama M, Kikuchi M, Natsukawa N, Shinobu N, Imaizumi T, et al (2004) The RNA helicase RIG1 has an essential function in double-stranded RNA-induced innate antiviral responses. Nat Immunol 5: 730–737. 15208624 10.1038/ni1087

[2] Kawai T, Akira S (2006) Innate immune recognition of viral infection. Nat Immunol 7: 131–137. 16424890 10.1038/ni1303

[3] Saha SK, Pietras EM, He JQ, Kang JR, Liu SY, et al (2006) Regulation of antiviral responses by a direct and specific interaction between TRAF3 and Cardif. EMBO J 25: 3257–3263. 16858409 10.1038/sj.emboj.7601220 PMC1523175

[4] Sasai M, Shingai M, Funami K, Yoneyama M, Fujita T, et al (2006) NAK-associated protein 1 participates in both the TLR3 and the cytoplasmic pathways in type I IFN induction. J Immunol 177: 8676–8683. 17142768 10.4049/jimmunol.177.12.8676

[5] Luo D, Ding SC, Vela A, Kohlway A, Lindenbach BD, et al (2011) Structural insights into RNA recognition by RIG1. Cell 147: 409–22. 22000018 10.1016/j.cell.2011.09.023 PMC3222294

[6] Wu B, Peisley A, Richards C, Yao H, Zeng X, et al (2013) Structural basis for dsRNA recognition, filament formation, and antiviral signal activation by MDA5. Cell 152: 276–89. 23273991 10.1016/j.cell.2012.11.048

[7] Loo YM, Gale M (2011) Immune Signaling by RIG1-like Receptors. Immunity 34: 680–692. 21616437 10.1016/j.immuni.2011.05.003 PMC3177755

[8] Takahasi K, Kumeta H, Tsuduki N, Narita R, Shigemoto T, et al (2009) Solution structure of cytosolic RNA sensor MDA 5 and LGP 2 C-terminal domains: identification of the RNA recognition loop in RIG1-like receptors. J Biol Chem 284: 17465–17474. 19380577 10.1074/jbc.M109.007179 PMC2719387

[9] Saito T, Hirai R, Loo YM, Owen D, Johnson CL, et al (2007) Regulation of innate antiviral defenses through a shared repressor domain in RIG1 and LGP2. Proc Natl Acad Sci USA 104: 582–587. 17190814 10.1073/pnas.0606699104 PMC1766428

[10] Kowalinski E, Lunardi T, McCarthy AA, Louber J, Brunel J, et al (2011) Structural basis for the activation of innate immune pattern-recognition receptor RIG1 by viral RNA. Cell 147: 423–35. 22000019 10.1016/j.cell.2011.09.039

[11] Cui S, Eisena Cher K, Kirchhofer A, Brzozka K, Lammens A, et al (2008) The C-terminal regulatory domain is the RNA 5′-triphosphate sensor of RIG1. Mol Cell 29: 169–179. 18243112 10.1016/j.molcel.2007.10.032

[12] Peisley A, Lin C, Wu B, Orme-Johnson M, Liu M, et al (2011) Cooperative assembly and dynamic disassembly of MDA5 filaments for viral dsRNA recognition. Proc Natl Acad Sci USA 108: 21010–21015. 22160685 10.1073/pnas.1113651108 PMC3248507

[13] Kato H, Takeuchi O, Mikamo-Satoh E, Hirai R, Kawai T, et al (2008) Length-dependent recognition of double-stranded ribonucleic acids by retinoic acid-inducible gene-I and melanoma differentiation-associated gene 5. J Exp Med 205: 1601–10. 18591409 10.1084/jem.20080091 PMC2442638

[14] Kato H, Takeuchi O, Sato S, Yoneyama M, Yamamoto M, et al (2006) Differential roles of MDA5 and RIG1 helicases in the recognition of RNA viruses. Nature 441: 101–105. 16625202 10.1038/nature04734

[15] Kato H, Takahasi K, Fujita T (2011) RIG1-like receptors: cytoplasmic sensors for non-self RNA. Immunol Rev 243: 91–98. 21884169 10.1111/j.1600-065X.2011.01052.x

[16] Triantafilou K, Vakakis E, Kar S, Richer E, Evans GL, et al (2012) Visualisation of direct interaction of MDA5 and the dsRNA replicative intermediate form of positive strand RNA viruses. J Cell Sci 125: 4761–4769. 22797917 10.1242/jcs.103887

[17] Baum A, Sachidanandam R, Garcia-Sastre A (2010) Preference of RIG1 for short viral RNA molecules in infected cells revealed by next by next-generation sequencing Proc Natl Acad Sci USA. 107: 16303–16308. 10.1073/pnas.1005077107 PMC2941304 20805493

[18] Schlee M, Roth A, Hornung V, Hagmann CA, Wimmenauer V, et al (2009) Recognition of 5′ triphosphate by RIG1 helicase requires short blunt double-stranded RNA as contained in panhandle of negative-strand virus. Immunity 31: 25–34. 19576794 10.1016/j.immuni.2009.05.008 PMC2824854

[19] Komuro A, Horvath CM (2006) RNA- and virus-independent inhibition of antiviral signaling by RNA helicase LGP2. J Virol 80: 12332–12342. 17020950 10.1128/JVI.01325-06 PMC1676302

[20] Rothenfusser S, Goutagny N, DiPerna G, Gong M, Monks BG, et al (2005) The RNA helicase LGP2 inhibits TLR-independent sensing of viral replication by retinoic acid-inducible gene-I. J Immunol 175: 5260–5268. 16210631 10.4049/jimmunol.175.8.5260

[21] Kang DC, Gopalkrishnan RV, Lin L, Randolph A, Valerie K, et al (2004) Expression analysis and genomic characterization of human melanoma differentiation associated gene-5, MDA5: a novel type I interferon-responsive apoptosis-inducing gene. Oncogene 23: 1789–1800. 14676839 10.1038/sj.onc.1207300

[22] Chang M, Collet B, Nie P, Lester K, Campbell S, et al (2011) Expression and functional characterization of the RIG1-like receptors MDA5 and LGP2 in Rainbow trout (*Oncorhynchus mykiss*). J Virol 85: 8403–12. 21680521 10.1128/JVI.00445-10 PMC3147945

[23] FAO (2000) Water Buffalo: an asset undervalued, FROfAa Bangkok, Thailand: Pacific Editor: 1–6.

[24] Borriello G, Capparelli R, Bianco M, Fenizia D, Alfano F, et al (2006) Genetic resistance to *Brucella abortus* in the water buffalo (*Bubalus bubalis*). Infect Immun 74: 2115–2120. 16552040 10.1128/IAI.74.4.2115-2120.2006 PMC1418909

[25] Livak KJ, Schmittgen TD (2001) Analysis of relative gene expression data using real-time quantitative PCR and the 2^−ΔΔCt^ method. Methods 25: 402–408. 11846609 10.1006/meth.2001.1262

[26] Hess B, Kutzner C, van der Spoel D, Lindahl E (2008) GROMACS 4: algorithms for highly efficient, load-balanced, and scalable molecular simulation. J Chem Theory Comput 14: 435–447. 10.1021/ct700301q 26620784

[27] Humphrey W, Dalke A, Schulten K (1996) VMD: visual molecular dynamics. J Mol Graph 14: 33–38. 8744570 10.1016/0263-7855(96)00018-5

[28] Spiliotopoulos D, Spitaleri A, Musco G (2012) Exploring PHD fingers and H3K4me0 interactions with molecular dynamics simulations and binding free energy calculations: AIRE-PHD1, a comparative study. PLoS One 7: e46902. 23077531 10.1371/journal.pone.0046902 PMC3471955

[29] Tamura K, Peterson D, Peterson N, Stecher G, Nei M, et al (2011) MEGA5: Molecular Evolutionary Genetics Analysis using Maximum Likelihood, Evolutionary Distance, and Maximum Parsimony Methods. Mol Biol Evol 28: 2731–2739. 21546353 10.1093/molbev/msr121 PMC3203626

[30] Kato H, Sato S, Yoneyama M, Yamamoto M, Uematsu S, et al (2005) Cell type-specific involvement of RIG-I in antiviral response. Immunity 23: 19–28. 16039576 10.1016/j.immuni.2005.04.010

[31] Slater L, Bartlett NW, Haas JJ, Zhu J, Message SD (2010) Co-ordinated role of TLR3, RIG1 and MDA5 in the innate response to rhinovirus in bronchial epithelium. PLoS Pathog 6: e1001178. 21079690 10.1371/journal.ppat.1001178 PMC2973831

[32] Bartenschlager R, Pietschmann T (2005) Efficient hepatitis C virus cell culture system: what a difference the host cell makes. Proc Natl Acad Sci USA 102: 9739–9740. 15998731 10.1073/pnas.0504296102 PMC1175013

[33] Eisenacher K, Krug A (2012) Regulation of RLR-mediated innate immune signaling–it is all about keeping the balance. Eur J Cell Biol 91: 36–47. 21481967 10.1016/j.ejcb.2011.01.011

[34] Biacchesi S, LeBerre M, Lamoureux A, Louise Y, Emilie Lauret E, et al (2009) Mitochondrial antiviral signaling protein plays a major role in induction of the fish innate immune response against RNA and DNA Viruses. J Virol 83: 7815–7827. 19474100 10.1128/JVI.00404-09 PMC2715792

[35] Huang S, Yuan S, Guo L, Yu Y, Li J, et al (2008) Genomic analysis of the immune gene repertoire of amphioxus reveals extraordinary innate complexity and diversity. Genome Res 18: 1112–1126. 18562681 10.1101/gr.069674.107 PMC2493400

[36] Sarkar T, Desalle R, Fisher PB (2008) Evolution of MDA5/RIG1-dependent innate immunity: independent evolution by domain grafting. Proc Natl Acad Sci USA 105: 17040–17045. 18971330 10.1073/pnas.0804956105 PMC2579374

[37] Zou J, Chang M, Nie P, Secombes CJ (2009) Origin and evolution of the RIG1 like RNA helicase gene family. BMC Evol Biol 9: 85. 19400936 10.1186/1471-2148-9-85 PMC2686710

[38] Hibino T, Loza-Coll M, Messier C, Majeske AJ, Cohen AH, et al (2006) The immune gene repertoire encoded in the purple sea urchin genome. Dev Biol 300: 349–365. 17027739 10.1016/j.ydbio.2006.08.065

[39] Jiang F, Ramanathan A, Miller MT, Tang GQ, Gale M Jr, et al (2011) Structural basis of RNA recognition and activation by innate immune receptor RIG-1. Nature 479: 423–427. 21947008 10.1038/nature10537 PMC3430514

[40] Civril F, Bennett M, Moldt M, Deimling T, Witte G, et al (2011) The RIG1 ATPase domain structure reveals insights into ATP-dependent antiviral signaling. EMBO Rep 12: 1127–34. 21979817 10.1038/embor.2011.190 PMC3207106

[41] Lu C, Xu H, Ranjith-Kumar CT, Brooks MT, Hou TY, et al (2010) The structural basis of 5′ triphosphate double-stranded RNA recognition by RIG1 C-terminal domain. Structure 18: 1032–43. 20637642 10.1016/j.str.2010.05.007 PMC2919622

[42] Wang Y, Ludwig J, Schuberth C, Goldeck M, Schlee M, et al (2010) Structural and functional insights into 5′-ppp RNA pattern recognition by the innate immune receptor RIG1. Nat Struct Mol Biol 7: 781–787. 10.1038/nsmb.1863 PMC3744876 20581823

[43] Lech M, Ferrufino AA, Skuginna V, Susanti HE, Anders HG (2010) Quantitative expression of RIG-like helicase, NOD-like receptor and inflammasome-related mRNAs in humans and mice. Int Immunol 22: 717–728. 20584763 10.1093/intimm/dxq058

[44] Ohtani M, Hikima J, Kondo H, Hirono I, Jung TS, et al (2011) Characterization and antiviral function of a cytosolic sensor gene, MDA5, in Japanese flounder, *Paralichthys olivaceus* . Dev Comp Immunol 35: 554–562. 21185857 10.1016/j.dci.2010.12.013

[45] Venkataraman T, Valdes M, Elsby R, Kakuta S, Caceres G, et al (2007) Loss of DExD/H box RNA helicase LGP2 manifests disparate antiviral responses. J Immunol 178: 6444–6455. 17475874 10.4049/jimmunol.178.10.6444

[46] Haller O, Kochs G, Weber F (2006) The interferon response circuit: induction and suppression by pathogenic viruses. Virol 344: 119–130. 10.1016/j.virol.2005.09.024 PMC7125643 16364743

[47] Castanier C, Zemirli N, Portier A, Garcin D, Bidere N, et al (2012) MAVS ubiquitination by the E3 ligase TRIM25 and degradation by the proteasome is involved in type I interferon production after activation of the antiviral RIG1-like receptors. BMC Biol 10: 44. 22626058 10.1186/1741-7007-10-44 PMC3372421

[48] Xing F, Matsumiya T, Onomoto K, Hayakari R, Imaizumi T (2012) Foreign RNA induces the degradation of mitochondrial antiviral signaling protein (MAVS): the role of intracellular antiviral factors. PLoS ONE 7: e45136. 23028806 10.1371/journal.pone.0045136 PMC3444469

[49] Sweeney SE, Kimbler TB, Firestein GS (2010) Synoviocyte innate responses: II Pivotal role of interferon regulatory factor 3. J Immunol 184: 7162–7168. 20483755 10.4049/jimmunol.0903944 PMC2913682

[50] Sun Q, Sun L, Liu HH, Chen X, Seth RB, et al (2006) The specific and essential role of MAVS in antiviral innate immune responses. Immunity 24: 633–642. 16713980 10.1016/j.immuni.2006.04.004

[51] Bustos-Arriaga J, García-Machorro J, León-Juárez M, García-Cordero J, Santos-Argumedo L, et al.. (2011) Activation of the innate immune response against DENV in normal non-transformed human fibroblasts. PLoS Negl Trop Dis 5, e1420 doi:101371/journalpntd0001420. 10.1371/journal.pntd.0001420 PMC3243703 22206025

[52] Li Y, Li C, Xue P, Zhong B, Mao AP, et al (2009) ISG56 is a negative-feedback regulator of virus-triggered signaling and cellular antiviral response. Proc Natl Acad Sci USA 106: 7945–7950. 19416887 10.1073/pnas.0900818106 PMC2683125

