# Supplementary material for: Insight into Buffalo (Bubalus bubalis) RIG1 and MDA5 Receptors: A Comparative Study on dsRNA Recognition and In-Vitro Antiviral Response
Source: PLoS One. 2014 Feb 26;9(2):e89788. doi: 10.1371/journal.pone.0089788 (PMC3935933; doi:10.1371/journal.pone.0089788)
Supplement: File S1 — Supporting Text, Tables, and Figures. Text S1: Methodology for isolation and culture of buffalo foetal fibroblast cells. Text S2: Procedures for model construction and simulation analysis. Text S3: Deduced amino acid sequence of buffalo RIG1 and MDA5. Table S1: PCR cycling parameters used for the amplification of RLR genes. Table S2: Sequences (NCBI Accession numbers) used for phylogenetic analysis. Table S3: Comparative analysis of the stereo-chemical parameters of RIG1, MDA5 and their corresponding templates. Figure S1: Agarose gel electrophoresis of amplified products of different fragments of buffalo RIG1 and MDA5 genes. Figure S2: Overall tertiary structure of helicase and C-terminal domains of (A) RIG1 and (B) MDA5 proteins of buffalo. The helicases and C terminal domains of buffalo RIG1 and MDA5 were modeled based on the X-Ray crystallographic structures of human RIG1 and MDA5 (termed as templates). The pair-wise alignment of buffalo and human sequences showed 82 and 84 percent sequence identities for RIG1 and MDA5, respectively. Due to this striking homology between target and template proteins, the built models retained all the key structural features of the templates that included HEL-1, HEL-2, HEL-2i, and the C-terminal domains. Figure S3: Ramachandran plot of buffalo (A) RIG1 and (B) MDA5 receptors. Stereochemical qualities of modeled proteins were found to be highly comparable to those of templates, indicating quality of the models were reasonably good to carry out further studies. Figure S4: Stability parameters of RIG1 and MDA5 receptors as a function of simulation time. (A) RMSD of RIG1. (B) RMSD of MDA5. (C) RMSF of RIG1. (D) RMSF of MDA5. In each panel, red color indicates stability parameters of dsRNA bound receptors and black color denotes receptors without dsRNA. Figure S5: Western analysis of intestinal tissue to determine specificity and cross reactivity of primary antibodies to RIG1 and MDA5. Dilution of primary antibodies 1∶500; Dilution of [file pone.0089788.s001.pdf]

Supporting Information

Insight into Buffalo (*Bubalus bubalis*) RIG1 and MDA5 receptors: A comparative study on dsRNA recognition and *in-vitro* antiviral response

Manvender Singh, Biswajit Brahma, Jitendra Maharana, Mahesh Chandra Patra, Sushil Kumar, Purusottam Mishra, Megha Saini, Bidhan Chandra De, Sourav Mahanty, Tirtha Kumar Datta, Sachinandan De*

Authors' affiliations: Animal Genomics Lab, Animal Biotechnology Center, National Dairy Research Institute, Karnal, Haryana 132001. India

***Corresponding author:** Sachinandan De, Animal Genomics Lab, Animal Biotechnology Center, National Dairy Research Institute, Karnal-132001, Haryana, India.
E-mail: sachinandan@ndri.res.in. Tel.: +91-1842259503. Fax +91-1842250042.

1. Supporting Methods

Table S1: PCR cycling parameters used for the amplification of RLR genes.

Step	Process	Temp/time
1	Initial Denaturation	94^0C for 2 min
2	Denaturation	94^0C for 45 sec
3	Annealing	63^0C for 30 sec $\Delta t = -1^0$C/cycle (Decrease annealing temperature by 1^0C / cycle)
4	Extension	68^0C for 30 sec
5	Repeat steps 2 to 4 (5 cycles).	
6	Denaturation	94^0C for 45 sec
7	Annealing	58^0C for 30 sec
8	Extension	68^0C for 30 sec
9	Repeat steps 6 to 8 (35 cycles).	
10	Extension	68^0C for 5 min
11	Holding	4^0C forever

Text S1: Methodology for isolation and culture of buffalo foetal fibroblast cells.

Preparation of culture media

Complete Dulbecco Modified Eagle's medium (DMEM) was prepared as per manufacturer's guidelines, and supplemented with 25mM HEPES, 10ng/ml EGF and L-Glutamine (2 mM). The pH of the medium was then adjusted to 7.4. Penicillin-G and streptomycin sulphate were added to final concentration of 10000U/ml and 100 µg/ml, respectively. Foetal bovine serum (FBS) was added to final concentration of 10 percent. The medium was then sterilized by passing it through 0.22 µm filter unit.

Isolation and culturing of foetal fibroblasts

Sterile techniques were used during all cell isolation procedures, all of which were performed under laminar hood at room temperature unless otherwise stated. Gravid uteri (2-3 months of pregnancy)

were obtained from abattoir house, New Delhi Municipal Corporation. The foetus was removed from uterus aseptically. A small section of skin tissue was removed from foetus and cut into small pieces (approximately 2-3 mm x 2-3 mm) by scalpel and forceps. The tissue pieces were then washed first in 70 percent ethanol and then in sterile phosphate buffer saline (PBS) solution. The pieces were then placed onto 35 x 10 mm Nunclon surface tissue culture disk (Nunc, Roskilde, Denmark) and passed rapidly and carefully through the Bunsen flame in order facilitate adherence of minced tissue pieces to the plastic surface, but so as not to heat-damage the minced tissue. Complete DMEM medium was added carefully without disturbing the tissue. The culture plates were then maintained in a humidified atmosphere of 5 percent CO_2 and 95 percent air at a temperature of 37^0C in an Innova CO-170 water jacketed CO_2 incubator (New Brunswick Scientific Co., Inc., Edison, NJ, USA). The fibroblasts started to grow from the minced fragments in 2-3 days. When there are sufficient cells, they are detached enzymatically with trypsin-EDTA solution and plated in Petri dishes, or 75 cm^2 culture flasks, for proliferation. For maintenance, the medium of the cultured cells was replaced after every two days. The general morphology, growth of cell population, and the presence of any microbial contaminants were checked regularly under an inverted microscope in phase contrast. Dishes or flasks with cells at about 70 percent confluence were treated with trypsin-EDTA; the cells were then harvested and either frozen or divided for further proliferation. For dishes with non-confluent cells the medium is discarded and replaced with fresh medium.

Table S2: Sequences (NCBI Accession numbers) used for phylogenetic analysis

Species	RIG1	MDA5
Bos taurus	XM_580928.6	XM_615590.5
Ovis aries	XM_004005323.1	XM_004004655
Sus scrofa	NM_213804.2	NM_001100194
Felis catus	XM_003995540.1	XM_003990812.1
Homo sapiens	NM_014314.3	NM_022168.3
Equus caballus	XM_001497845.2	XM_001494330.1
Mus musculus	NM_172689.3	NM_001164477.1
Macaca mulatta	NM_001042668.1	XXXXXXXXX
Taeniopygia guttata	XM_002194524.2	XXXXXXXXX
Xenopus laevis	XM_002935671.2	XM_002933274.2
Gallus gallus	XXXXXXXXXXX	NM_001193638.1
Pan troglodytes	XXXXXXXXXXX	XM_003820935
Bubalus bubalis	KF517376	KF517377

Text S2: Procedures for model construction and simulation analysis

Homology Modeling

The amino acid sequences of buffalo RIG1 and MDA5 were deduced from respective nucleotide sequences obtained in this study. The homologous templates for model building were selected employing Domain Enhanced Lookup Time Accelerated (DELTA) BLAST [1] search against the known protein structures available in PDB. Based on maximum identity, X-ray crystallographic structures of Human RIG1 (PDB ID: 3TMI) [2] and MDA5 (PDB ID: 4GL2) [3] were selected as best templates for constructing three dimensional models for buffalo RIG1 and MDA5 receptors, respectively. The tertiary structures were predicted using advance modeling protocol of MODELLER 9.11 [4]. The resultant models were ranked based on Discrete Optimized Potential Energy (DOPE) score and the models with lowest DOPE scores were selected for further study. Stereo-chemical quality assessment and model validation were performed by PROCHECK [5],

ERRAT [6], and WHATIF [7] programs.

Principal component analysis

Principal component analysis (PCA) was performed by constructing a covariance matrix containing dominant low-frequency, large scale motions of main chain atoms of the amino acid residues along the MD trajectory. The matrix was then diagonalized to give rise eigenvectors and their corresponding eigenvalues. Normally, the first eigenvector represents the largest contribution to the global fluctuation of the system followed by the second eigenvector, and so on.

Binding free energy calculation

The binding free energy (ΔG_{bind}) was calculated by GMXAPBS tool, which uses Molecular Mechanics /Poisson Boltzmann Surface Area (MM/PBSA) method as follows:

$$\Delta G_{bind} = \langle G_{complex} - G_{protein} - G_{ligand} \rangle \qquad (1)$$

Where, $G_{complex}$, $G_{protein}$ and G_{ligand} are the free energies of complex, protein and ligand, respectively. The brackets indicate that the average of snapshots was taken from a single MD trajectory.

The free energy of each state was calculated as follows:

$$\langle G \rangle = \langle E_{MM} \rangle + \langle G_{PB} \rangle + \langle G_{SA} \rangle - T\langle S_{MM} \rangle \qquad (2)$$

$T < S_{MM}>$ is the entropic contribution of the solute, which was not evaluated in this study due to high computational cost and often this can produce unpredictable results.

G_{PB} and G_{SA} are the contributions from polar and nonpolar terms of the free energy of solvent continuum, the former is calculated via Poisson-Bolztmann equation and the latter is calculated as follows:

$$G_{SA} = \gamma \, SASA + \beta \qquad (3)$$

Where, γ is the surface tension proportionality constant, β is the free energy of nonpolar solvation for a point solute and SASA is the solvent accessible surface area. E_{MM} is the molecular mechanical energy, calculated as the sum of different contributions as follows:

$$E_{MM} = E_{vdW} + E_{ele} + E_{int} \qquad (4)$$

E_{vdW}, E_{ele} are the van der Waals (LJ) and the electrostatic energies, respectively. E_{int} is the internal energy including bond, angle and torsional angle energies. It is worth noting that in the case of single-trajectory experiments, the variation of E_{int} (E_{int}) equals zero in calculating the binding free energy according to eqn.1, since the internal energies of the complex and the separated parts (protein and ligand) are calculated from the same trajectory [8].

2. Supporting Results

Figure S1: Agarose gel electrophoresis of amplified products of different fragments of buffalo RIG1 and MDA5 genes.

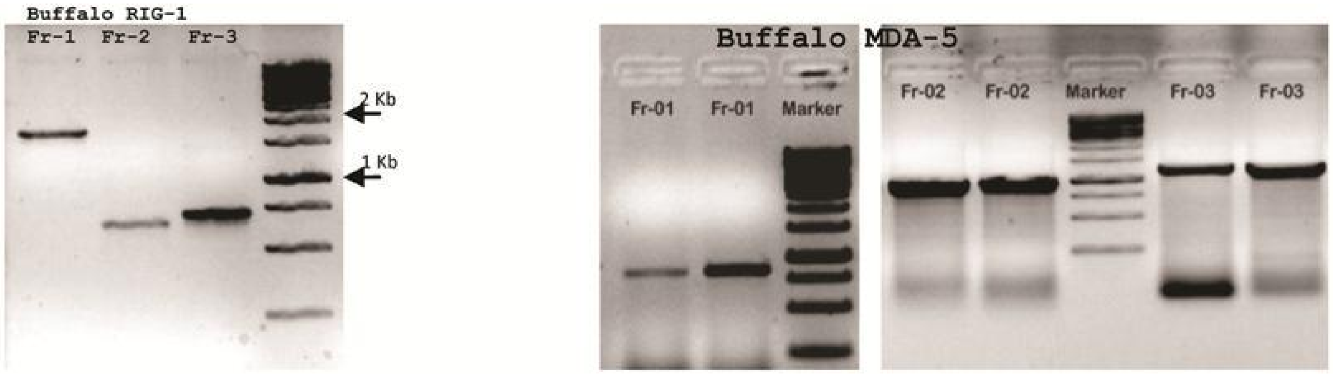

Text S3: Deduced amino acid sequence of buffalo RIG1 and MDA5

>Buffalo RIG1 (933 amino acids)

MTAEQRRNLHAFRDYVRKILDPTYILSYMTPWFRDDVVQHIQAEKNNKGPMEAASLFLQVLLELQ
EEGWFRGFLDALQQAGYSGLYEAIESWDFQKIEKLEEYRLLLKRLQPEFKTTINPEDILPEISGC
LINQECEEIIQISSNKGLMAGAEKMVECLLRSDKENWPKTLKLALEKEESKFSELWMVEKGAENV
QMKDLEDDEMKTLDVHIVYKEEPENQNRSQNSCSSSEVPPTYSPLKPRNYQLELALPAQKGKNTI
ICAPTGCGKTFVSLLICEHHLKRFPQGRKGKVVLFAVQVPLYEQQKSVFSEYFERFGYKVSGISG
ATADSISAEQIVENNDIIILTPQILVNSLKDGTIPSLSIFTLMIFDECHNTNKHHPYNMIMFHYL
DQKLGGSSDSLPQVIGLTASVGVGDAKNTAEATEYICKLCASLDTSVVATVRDNLEELEEVVYKP
QKFFRKVESRTTDRFKHIISQLMAETEALAKSIFEELGTVTLENLSQIQNRHFGTQKYEQWIIAV
QKACIVFQMPDKEEESRICKALFLYTSHLRKYNDALIINEDARMKDALNYLKNFFKNVRAAGFDA
IEQDLTQRFEEKLLELEGISMDPSNENAKLKDLCFILQEEYHLNPETRTILFVKTRALVDALKNW
IEENPKLSFLKPGILTGRGRTNQTMGMTLPAQKCALDAFRTNRDSKILIATSVADEGIDIAQCNL
VILYEYVGNVIKMIQTRGRGRARGSKCFLLTSNADVIEKEKLNICKEKMMNESISRLQGWNEAVF
KEKIRQIQIQEKLIRDNQGKVKPVVDKKNKKLLCGKCKTFACYTADIRVVEECHFTVVRDAFREC
FVTKLHPRPKKFGSFEKKAKIFCARKDCLHDWGIHVKYKTFEIPVIKIESFVVEDVATGAQTLYA
KWKDFNFEKIPFDAAEMSPWAQE

>Buffalo MDA5 (1021 amino acids)

```
MSSDGSSTDKNFCYLISCFRARVKRYIQVEPVLDYLTFLSPEVKEHIQRTAATTGDIQAADLLLDT
LERGNWPLGWARMFVEALRQAGNPLAARYVNPELTDLPSPSFENTHDECLQLLNLLQPTLVDKLLV
ADVLDKCVEEKLLTIEDRNRVSAAENNGNEAGVRELLKRIVQKENWFSTFLTILRQTGNDELAREL
TGTDCCEGNTESENLSQEDGLEVKEPLLLATDQPNLEVLDIENSSLESSFADSSIVSESDTSLAEG
SVSCLDESLGHNSNMGSDSGTMGSDSDDENVAQRASPEPELNLRPYQLEVAQPALEGKNIIICLPT
GSGKTRVAVYIAKDHLDKKKASEPGKVMVLVNKVPLVEQLFRKEFKPFLKKWYHVTRLSGDTQLKI
TFPEVVKSHDVIISTAQILENSLLNSEEGEDDGIELSDFSLIIIDECHHTNKEAVYNNIMRRYLKQ
KLKNNKLKKENKPVIPLPQIVGLTASPGVGGAKKQAKAEEHILKICANLDAVTIKTVKQNINLLKE
QIKEPCKKFVIADDTKKDPFKDKLLEIMTKIQTFCQINPMSDFGTQPYEQWLIQMEKKAAKEGNRK
DRVCAEHLRKYNEALQINDTIRMIDAYNHLETFYNDEKEKKFAVLLGDDSDESGDNGDDDAGDGKA
PLKLHETDDFLISLFLGNKKKLKKLAQNPEHENEKLIKLRNTIMEQYSRTEGSARGIIFTKTRQSA
YALSQWIIENEKFSEVGVKAHHLIGAGHSSEFKPMTQNEQKEVISKFRTGKINLLIATTVAEEGLD
IKECNIVIRYGLVTNEIAMVQARGRARADESTYVLVAQSGSGVVERETVNDFREKMMYKAIDRVQN
MKPEEYAHKILELQMQSIMEKKMKTKRSIAKQFKDKPSLINFLCKNCGVPACSGEDIHVIEKMHHV
NMTPEFKKLYLVRGNKALQTMCVDYQTNGEIICNKCGQAWGTMMVHKGLDLPCLKIKNFVVVFQNN
LPKKQYKKWVELPITFPDLNYSEYCLFSDED
```

Figure S2: Overall tertiary structure of helicase and C-terminal domains of (A) RIG1 and (B) MDA5 proteins of buffalo. The helicases and C terminal domains of buffalo RIG1 and MDA5 were modeled based on the X-Ray crystallographic structures of human RIG1 and MDA5 (termed as templates). The pair-wise alignment of buffalo and human sequences showed 82 and 84 percent sequence identities for RIG1 and MDA5, respectively. Due to this striking homology between target and template proteins, the built models retained all the key structural features of the templates that included HEL-1, HEL-2, HEL-2i, and the C-terminal domains.

A

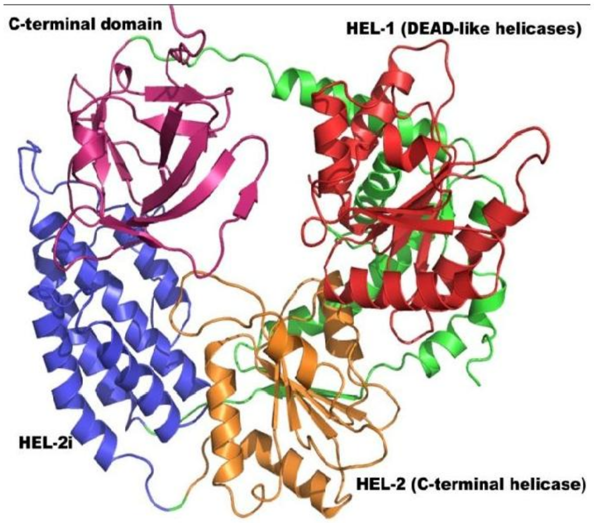

B

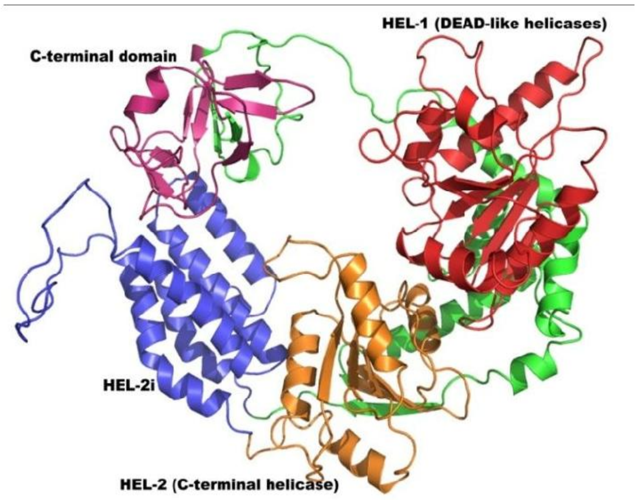

Figure S3: Ramachandran plot of buffalo (A) RIG1 and (B) MDA5 receptors. Stereochemical qualities of modeled proteins were found to be highly comparable to those of templates, indicating quality of the models were reasonably good to carry out further studies.

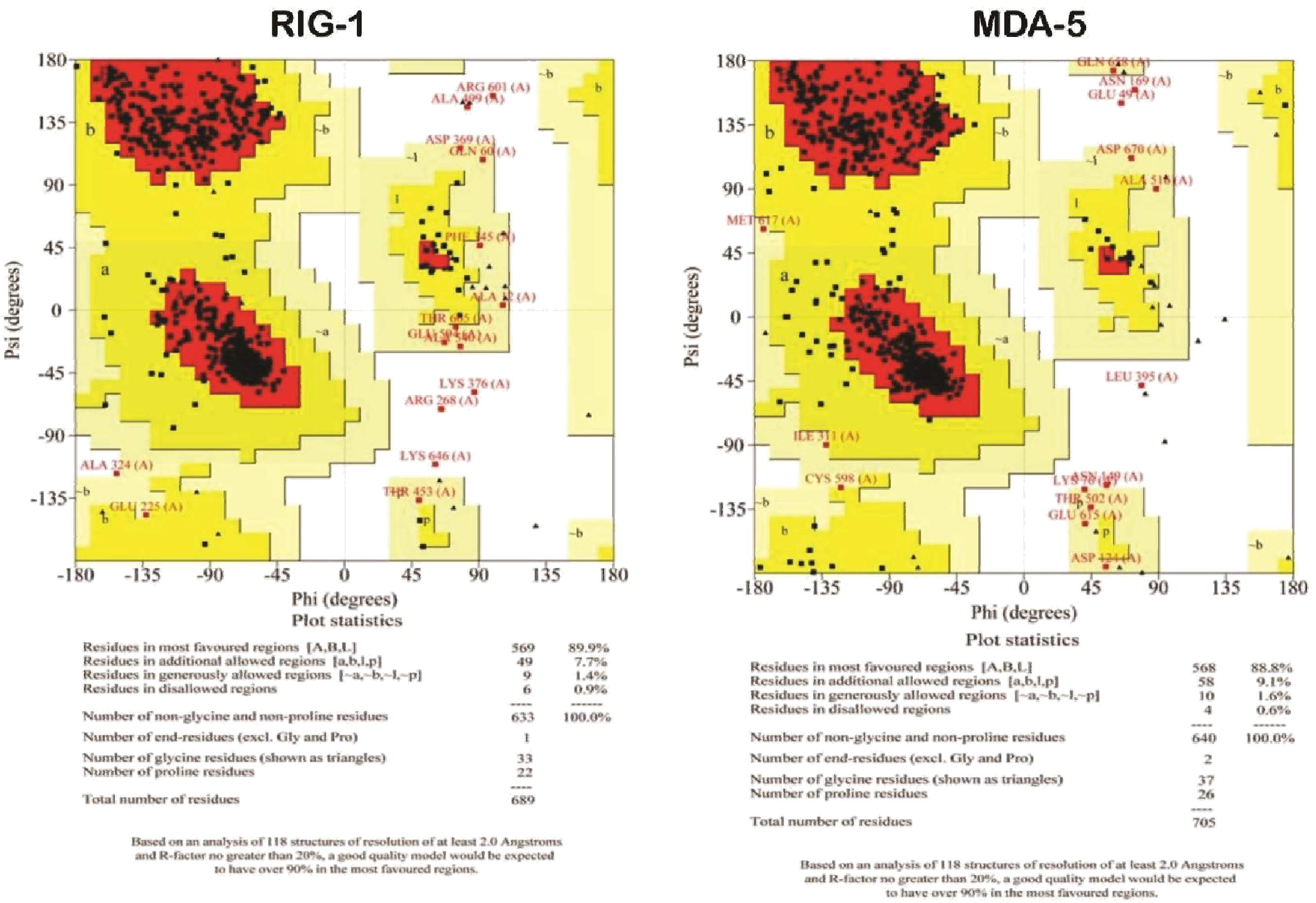

Table S3: Comparative analysis of the stereo-chemical parameters of RIG1, MDA5 and their corresponding templates.

**Servers**		**RIG1**	**[a]3TMI**	**MDA5**	**[b]4GL2**
**[c]Procheck**	Most favored regions (%)	89.4	84.1	88.0	94.0
	Additionally allowed Regions (%)	7.4	12.9	9.8	4.4
	Generously allowed Regions (%)	2.1	2.3	1.6	1.6
	Disallowed regions (%)	1.1	0.7	0.6	00
	[d]Overall G-factor	-0.09	-0.09	-0.09	0.11
**[e]Verify3D**	Averaged 3D-1D Score > 0.2	85.51	82.71	75.5	75.89
**[f]ERRAT**	Overall Quality	89.64	81.06	86.25	81.37
**[g]ProSA**	Z-Score	-10.62	-10.45	-10.45	-8.46
**[h]ProQ**	LG score	5.28	5.31	4.6	5.94
	MaxSub	0.46	4.49	0.44	0.49

[a]Template of RIG1

[b]Template of MDA5

[c]Procheck integrates Ramachandran plot for estimating accuracy of dihedral angles (Φ/Ψ). For an extremely good quality model 90% of the residues should be within most favored region.

[d]For a good quality model the G-factor should be above the cutoff value of -0.5 [9].

[e]% of residues complementing sequence to structure agreement. For a good quality model the Verify 3D score should be greater than 80% [10].

[f]Errat score provides accuracy of the nonbonded atoms, and a good quality model must have a score greater than the acceptable value of 50% [6]

[g]ProSA gives Z-score of a given model which should fall within range of protein structures of similar size available in PDB [11].

[h]ProQ analysis indicates that for a given model LGscore and MaxSub scores must be above 4.0 and 0.4, respectively [12].

Figure S4: Stability parameters of RIG1 and MDA5 receptors as a function of simulation time. (A) RMSD of RIG1. (B) RMSD of MDA5. (C) RMSF of RIG1. (D) RMSF of MDA5. In each panel, red color indicates stability parameters of dsRNA bound receptors and black color denotes receptors without dsRNA.

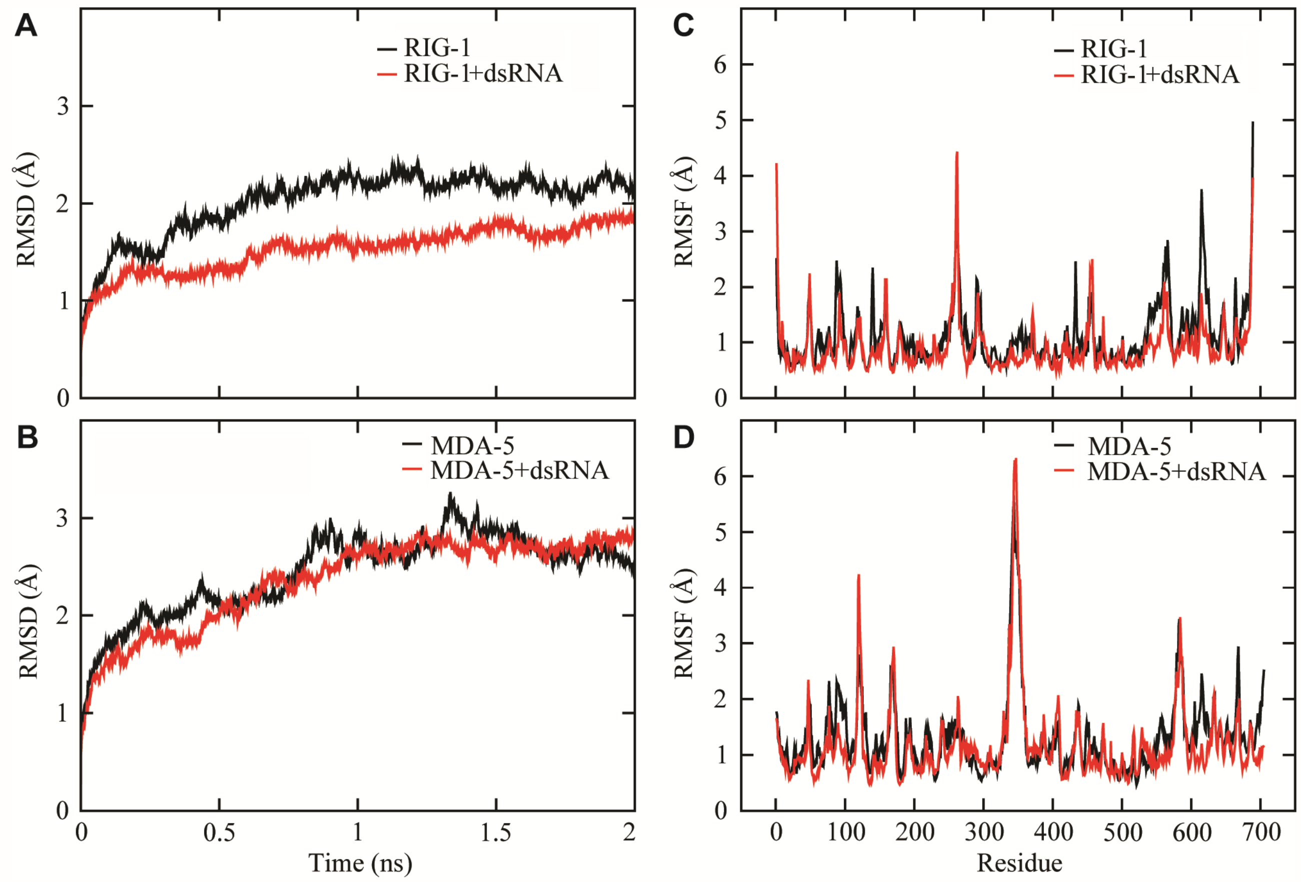

Figure S5: Western analysis of intestinal tissue to determine specificity and cross reactivity of primary antibodies to RIG1 and MDA5. Dilution of primary antibodies 1:500; Dilution of secondary anti goat IgG 1:50000. The chemiluminescence was detected on x-ray films using Immobilon Western Chemiluminescent HRP (Millipore Corporation, MA, USA) as substrate.

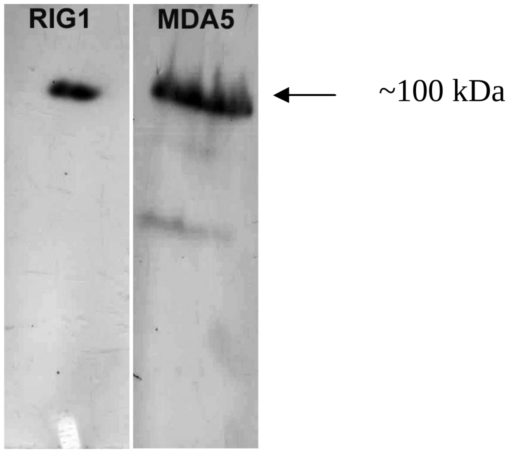

References:

1. Boratyn GM, Schäffer AA, Agarwala R, Altschul SF, Lipman DJ, et al. (2012) Domain enhanced lookup time accelerated BLAST. Biol Direct 7: 12.

2. Jiang F, Ramanathan A, Miller MT, Tang GQ, Gale M Jr, et al. (2011) Structural basis of RNA recognition and activation by innate immune receptor RIG1. Nature 479: 423-427.

3. Wu B, Peisley A, Richards C, Yao H, Zeng X, et al. (2013) Structural basis for dsRNA recognition, filament formation, and antiviral signal activation by MDA5. Cell 152: 276-289.

4. Fisher A, and Sali A (2003) Modeller: generation and refinement of homology-based protein structure models. Methods Enzymol 374: 461-491.

5. Laskowski RA, MacArthur MW, Moss DS, Thornton JM (1993) PROCHECK: a program to check the stereochemical quality of protein structures. J Appl Cryst 26: 283-291.

6. Colovos C and Yeates TO (1993) Verification of protein structures: patterns of non-bonded atomic interactions. Protein Sci 2:1511-1519.

7. Vriend G (1990) WHAT IF: a molecular modeling and drug design program. J Mol Graph 8: 52-56.

8. Huo S, Massova I, Kollman PA (2002) Computational alanine scanning of the 1:1 human growth hormone-receptor complex. J Comput Chem 23: 15-27.

9. Laskowski RA, MacArthur MW, Moss DS, Thornton JM (1993) PROCHECK: A program to check the stereochemical quality of protein structures. J Appl Cryst 26: 283-291.

10. Lüthy R, Bowie JU, Eisenberg D (1992) Assessment of protein models with three-dimensional

profiles. Nature 356: 83-85.

11. Wiederstein M, Sippl MJ (2007) ProSA-web: interactive web service for the recognition of errors in three-dimensional structures of proteins. Nucleic Acids Res 35: W407-410.

12. Wallner B, Elofsson A (2003) Can correct protein models be identified? Protein Sci 12:1073-86.
